# Outcomes of Bronchial Artery Embolization for Life-Threatening Hemoptysis Secondary to Tuberculosis

**DOI:** 10.1371/journal.pone.0115956

**Published:** 2014-12-26

**Authors:** Renguang Pei, Yunfeng Zhou, Guoxiang Wang, Heping Wang, Xinyu Huang, Xiaoxing Yan, Xiaohua Yang

**Affiliations:** 1 Department of Interventional Therapy, Yijishan Hospital of Wannan Medical College, Wuhu, P.R. China; 2 Department of Radiology, Yijishan Hospital of Wannan Medical College, Wuhu, P.R. China; Hospital San Agustín, Aviles, Asturias, Spain

## Abstract

**Objective:**

To appraise the immediate and long-term outcomes of bronchial arterial embolization for life-threatening hemoptysis secondary to tuberculosis.

**Methods:**

112 patients with life-threatening hemoptysis due to tuberculosis underwent bronchial artery embolization from January 2004 to February 2014. Life-threatening hemoptysis was defined as expectoration of at least 400 ml of blood in 24 hour. The median follow-up is 20 months, ranging from 2 to 52 months.

**Results:**

The hemoptysis control rate was 86.6% at 14 days, 84.8% at 30 days, 78.6% at 240 days, 75.9% at 360 days, respectively. None of these characteristics, including gender, age and tuberculosis status, was significantly associated with immediate control of bleeding. Patients with active tuberculosis had a significantly longer recurrence-free duration than did patients with inactive tuberculosis (P = 0.040), which was further confirmed by Cox regression hazards model (P = 0.046). There was no spinal cord complication or mortality related to bronchial artery embolization. The most common complication was transient chest pain.

**Conclusion:**

Bronchial arterial embolization is an effective and safe technique in the management of life-threatening hemoptysis secondary to tuberculosis. Active tuberculosis may be associated with a lower rate of recurrence of hemoptysis.

## Introduction

Hemoptysis is a relatively common presenting symptom in clinical practice, which can be a life-threatening respiratory emergency [1]. Large-volume hemoptysis with conservative management carries a mortality rate more than 50% [2], [3], [4]. Bronchial artery embolization (BAE) offers an effective and minimally invasive procedure for the management of life-threatening hemoptysis, as opposed to surgery and bronchoscopy [5].

BAE was first performed in 1973 [6]. However, the practice of BAE was fettered by the complications of spinal cord ischemia, which was caused by occlusion of spinal arteries that may arise from bronchial or intercostals arteries [7], [8]. It is not until the advent of a ‘super-selective’ technique in the mid 1990s that the practice of BAE was unfettered, which permits a safe embolization beyond the origin of the spinal artery with the use of microcatheter [9]. Since then, studies have been growing that assess the efficacy and safety of BAE in controlling hemoptysis secondary to diverse causes [10].

To date, sparse data are known about outcomes of BAE for hemoptysis secondary to tuberculosis. Meanwhile, these studies suffer either a relative small sample size or a relative short follow-up time [11], [12], [13], [14]. Available data on outcomes of BAE for hemoptysis mainly come from developed western populations, where bronchogenic carcinoma and bronchiectasis account for most hemoptysis cases [5]. Nevertheless, tuberculosis is the leading cause of hemoptysis in developing countries [5], such as in China, where the prevalence of tuberculosis is an estimated 442 cases per 100 000 population, accounting for 11% of global tuberculosis incidence [15]. Hence, we conducted a retrospective study to review our institution's 10 years experience in BAE for hemoptysis due to tuberculosis exclusively.

## Materials and Methods

### Study population

A total of 112 patients received BAE for life-threatening hemoptysis secondary to tuberculosis at the Yijishan Hospital between January 2004 and February 2014. Life-threatening hemoptysis was defined as expectoration of at least 400 ml of blood in 24 hour, in spite of the administration of intravenous vasopressin [16]. All patients were hospitalized and underwent standard medical management, including administration of intravenous vasopressin, correction of hypoxemia, correction of hemodynamic instability, and transfusion with blood products as necessary. The patients with active tuberculosis were given anti-tuberculosis regiment. Active tuberculosis was defined on the basis of acid-fast bacilli (AFB) positive, clinical suspicion or imaging including consolidation, endobronchial spread pattern, miliary pattern, or tree-in-bud opacities. Post-tuberculosis sequelae (Inactive tuberculosis) was defined on the basis of previous history of tuberculosis and AFB-negative with imaging including bronchiectasis, fibrosis, calcified nodules [17].

Follow-up information was retrospectively retrieved from inpatient and outpatient medical records or direct contact by telephone. In order to obtain the integrated and reliable data, two people independently reviewed the database of Department of Intervention and Radiology. The median follow-up was 20 months, ranging from 2 months to 52 months. For each patient, the end of follow-up was defined as the date of death or the last date when the patient was available for follow-up. There were a total of nine deaths during the follow-up period, two patients dying of chronic pulmonary hypertension, three patients dying of bacterial pneumonia, one patients dying of fungal septicemia, one patient dying of active multidrug-resistant tuberculosis, and two patients dying of recurrent hemoptysis.

Successful control of bleeding was defined as no hemoptysis or minimal hemoptysis after BAE. Immediate control of bleeding was defined as successful control of hemoptysis lasting more than 2 weeks after BAE [18]. Recurrent hemoptysis was defined as expectoration of>40 ml of blood in 24 hour after BAE. The decision to repeat BAE was based on the same instructions as the first BAE. Minor hemoptysis was managed conservatively. This study was approved by the Research and Ethics Committee of the Yijishan Hospital of Wannan Medical College with a written informed consent by all these patients.

### BAE procedure

All patients underwent BAE following a standard BAE protocol. The femoral artery was catheterized under local anaesthesia. Both bronchial artery and nonbronchial systemic arteries were opacified. In all cases, bronchial or nonbronchial arteries were found to be abnormal with extravasation of contrast medium, torturous hypertrophy, regions of hypervascularity, or systemic-to-pulmonic shunting. Pulmonary angiography was not performed in any patients because abnormalities of bronchial or nonbronchial arteries were found in all cases. Agents used for embolization were absorbable particles of gelatin sponge (gelfoam, Jinling Pharmaceutical Company Limited, China) with a range of sizes from 1 mm to 3 mm. Visualization of spinal artery was an absolute contraindication to the procedure unless a safe embolization beyond the origin of spinal artery could be ensured.

### Statistical analysis

The association between immediate control of bleeding and the clinical characteristics were evaluated by chi-squared test. The recurrent-free duration (RFD) was calculated by Kaplan-Meier method and log-rank test for univariate analysis. The Cox proportional hazards model was used for multivariate analysis. All of the statistical tests and P value were two-tailed and P values of <0.05 were considered significant. All analyses were performed using the SPSS 16.0 software (Chicago, USA).

## Results

### Patient characteristics

We retrospectively reviewed a cohort of 112 consecutive patients (98 males, 14 females) who underwent BAE secondary to hemoptysis, with an age range of 16–81 years (median, 57 years). Sixty eight patients had active tuberculosis, while forty four patients had post-tuberculosis sequelae (Inactive).

Abnormalities of bronchial or nonbronchial arteries were found in all patients. Angiograms showed multiple abnormalities in every patient. Systemic-to-pulmonary venous shunts occurred in 18 patients, contrast extravasation in 96 patients, torturous hypertrophy in 107 patients, hypervascularity in 82 patients. A bronchial or intercostals origin of spinal artery was not found in all these patients.

### Immediate Control of Bleeding

Immediate arrest of hemoptysis was obtained in 97 (86.6%) patients. Association of immediate control with clinical characteristics is shown in Table 1. None of these characteristics, including gender, age and tuberculosis status, was significantly associated with immediate control of bleeding.

**Table 1:** Immediate control of bleeding with clinical characteristics.

Characteristics	No.	Immediate Control (n/%)		*P*
		Yes	No	
**Gender**				0.916
Male	98	85 (86.7)	13 (13.3)	
Female	14	12 (85.7)	2 (14.3)	
**Age (years)**				0.291
≤50	53	44 (83.0)	9 (17.0)	
>50	59	53 (89.8)	6 (10.2)	
**Tuberculosis Status**				0.529
Active	68	60 (88.2)	8 (11.8)	
Inactive	44	37 (84.1)	7 (15.9)	

### Cumulative Recurrent-free Duration

**Figure 1:**
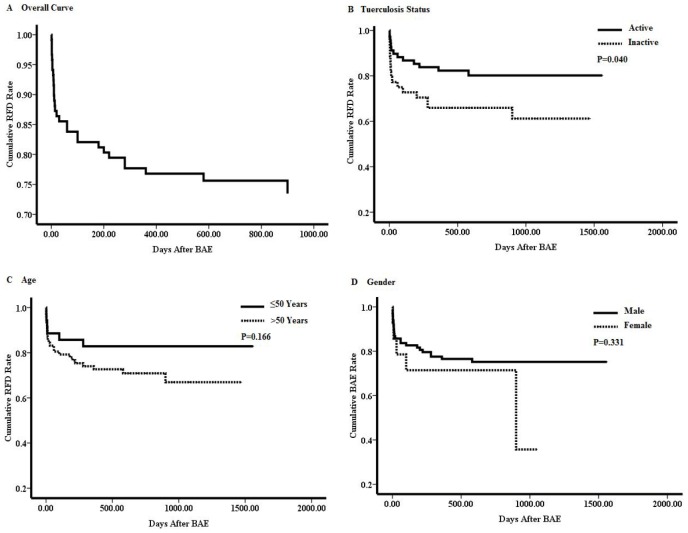
Hemoptysis recurrence-free duration (RFD) analyzed by Kaplan-Meier method.

The forepart of the Kaplan-Meier curve, which delineated the overall cumulative RFD rate, declined sharply, followed by a rear part of declining slowly (Fig. 1A). The overall cumulative RFD rate was 86.6% at 14 days, 84.8% at 30 days, 78.6% at 240 days, 75.9% at 360 days, respectively.

We further appraised association of RFD with clinical characteristics by univariate Kaplan-Meier analysis. Patients with active tuberculosis had a significantly higher RFD rate than did patients with inactive tuberculosis (Fig. 1B), whereas both of gender and age had no significant association with cumulative RFD rate (Fig. 1C and Fig. 1D). Multivariate analysis by Cox regression indicated that tuberculosis status served as a significant and independent prognostic factor (P = 0.048) in the context of this study (Table 2).

**Table 2:** Recurrence-free duration and clinical characteristics.

Characteristics	Univariate		Multivariate		
	Mean RFD ±SE(days)	Log-Rank *P*	Hazard Ration 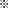	95% CI	*P*
**Gender**		0.331			0.308
Male	1199.587 (64.177)		1	Reference	
Female	706.500 (119.747)		1.670	0.632–4.476	
**Age**		0.166			0.132
≤50	1304.114 (95.353)		1	Reference	
>50	1050.678 (73.433)		1.998	0.811–4.921	
**Tuberculosis Status**		0.040			0.046
Active	1278.031 (70.497)		1	Reference	
Inactive	964.620 (101.148)		2.109	1.014–4.387	

**RFD: Recurrence-Free Duration; SE: Standard Error; CI: Confidence Interval.**

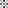
 **Estimated from Cox proportional hazards model.**

### Complications

BAE-related complications were not uncommon, and no major complications were observed (Table 3).

**Table 3:** BAE-related Complications.

Complication	No. (%)	Consequence
Chest pain	31 (27.7)	Transient		
Dysphagia	1 (0.9)	Transient		
Contrast reaction	3 (2.7)	Mild reaction was treated with dexamethasone		
Pulmonary infarct	0	_		
Bronchoesophageal fistula	0	_		
Paraplegia	0	_		
Mortality	0	_		

## Discussion

In the present study, we demonstrated that BAE was a safe and effective procedure for the management of life-threatening hemoptysis due to tuberculosis. Active tuberculosis was associated with a longer recurrence-free duration. BAE-related complications were not uncommon and transient, with no paraparesis observed.

The definition of life-threatening has not been completely agreed upon, generally relying on the volume of expectorated blood, which ranges from 100 ml to more than 1000 ml in different studies [16]. The eligibility criterion for entering our study was expectoration of at least 400 ml of blood in 24 hour. Patients who have hemoptysis inferior to 400 ml were excluded in this study. There has not yet been a consensus about the definition of recurrence of hemoptysis after BAE. Anuradha C defined recurrent hemoptysis as a total of>30 ml of bleeding in 24 hour [18], Shin BS defined recurrent hemoptysis as occurrence of frank hemoptysis [19]. Allowing for blood-tinged sputum, our study defined recurrent hemoptysis as expectoration of>40 ml of blood in 24 hour after BAE, relatively larger than the former criteria.

From the Kaplan-Meier curve delineating the overall cumulative RFD rate (Fig. 1A), the forepart falls sharply, suggesting that hemoptysis relapse mostly occurred in a relatively short time after BAE. This period may be too short, so that some clinical characteristics have no adequate time to influence tuberculosis progression, which can trigger hemoptysis recurrence. To some extent, this can explain why none of clinical characteristics was significantly associated with immediate control of bleeding in our study. Early recurrent bleeding, within the first weeks, may be attributed to incomplete embolization of the abnormal vessels [10]. The immediate hemoptysis control rate in our institution reaches 88.6%, which agrees with the previous reports, in which BAE resulted in an immediate arrest of bleeding in 86–90% of hemoptysis cases due to diverse causes [20], [21], [22].

Long-term recurrence rates vary depending on the underlying lung disease [5]. The underlying diseases for hemoptysis are numerous and vary in frequency depending upon population demographics [5]. Hemoptysis is largely attributed to tuberculosis in China, while bronchogenic carcinoma and chronic inflammatory lung diseases account for most cases in the western world [10]. High bleeding recurrence rates are observed in patients with aspergillosis or bronchial carcinoma [23], [24]. Recurrence rate approached 50% after BAE in hemoptysis cases secondary to bronchiectasis, aspergillosis or chronic bronchitis [24]. In our study, the overall cumulative recurrence-free rate was 84.8% at 30 days, 78.6% at 240 days, 75.9% at 360 days, respectively, which is similar to the results of these studies recruiting only tuberculosis patients in Korean population [19], [25], nevertheless, higher than the hemoptysis control rate of 51% at one year in Indian population [18].

When it comes to factors influencing long-term recurrence, our study showed active tuberculosis with anti-tuberculosis treatment functioned as a protective factor. This result of our study is consistent with those of previous reports [14], [19]. On the contrary, it has also been reported that active tuberculosis served as an increased risk of recurrence [13], [26]. The inconsistence among these reports may be due to patients either with multidrug-resistant tuberculosis or without receiving anti-tuberculosis regiment in the latter studies [13], [26]. BAE, as a palliative procedure for symptomatic control of hemoptysis, does not address the underlying disease process. Therefore, concurrent effective anti-tuberculosis therapy in patients with active tuberculosis may contribute to a longer recurrence-free duration [14], [19].

BAE-related complications in our institution were common, with a rate of 30% lower than that of 42% in a previous report [19], but higher than that of 13% and 4.9% in another previous reports [13], [27]. All complications of this present study were minor. Major complications, such as spinal cord ischemia, were observed in several studies [7], [8], [28], [29], but did not occur in our study. This is probably due to use of superselective embolization and no bronchial or intercostals origin of spinal artery found in our study.

This study may potentially be limited by its retrospective nature. However, independently reviewing the database of Department of Intervention and Radiology by two people in this study can help to obtain integrated and reliable data. Moreover, a total of 112 consecutive patients in a single institution can reduce bias from possible confounding factors.

In conclusion, our study has confirmed that BAE is a safe and effective treatment for the control of life-threatening hemoptysis secondary to tuberculosis. Active tuberculosis patients may carry a longer recurrence-free duration after BAE.
